# Supplementary material for: Glucose–Thymidine Ratio as a Metabolism Index Using 18F-FDG and 18F-FLT PET Uptake as a Potential Imaging Biomarker for Evaluating Immune Checkpoint Inhibitor Therapy
Source: Int J Mol Sci. 2022 Aug 17;23(16):9273. doi: 10.3390/ijms23169273 (PMC9409370; doi:10.3390/ijms23169273)
Supplement: Supplementary file 1 [file ijms-23-09273-s001.zip › ijms-1816390-supplementary.pdf]

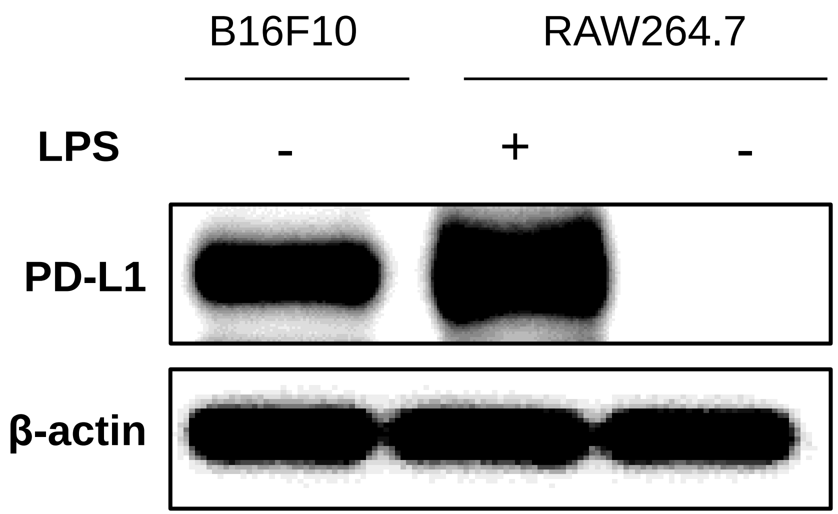

Figure S1: Western blot analysis was performed using RAW264.7 and mouse melanoma B16F10 following LPS treatment to confirm PD-L1 expression. PD-L1 expression was confirmed in LPS-treated RAW264.7 and similar expression was confirmed in B16F10.

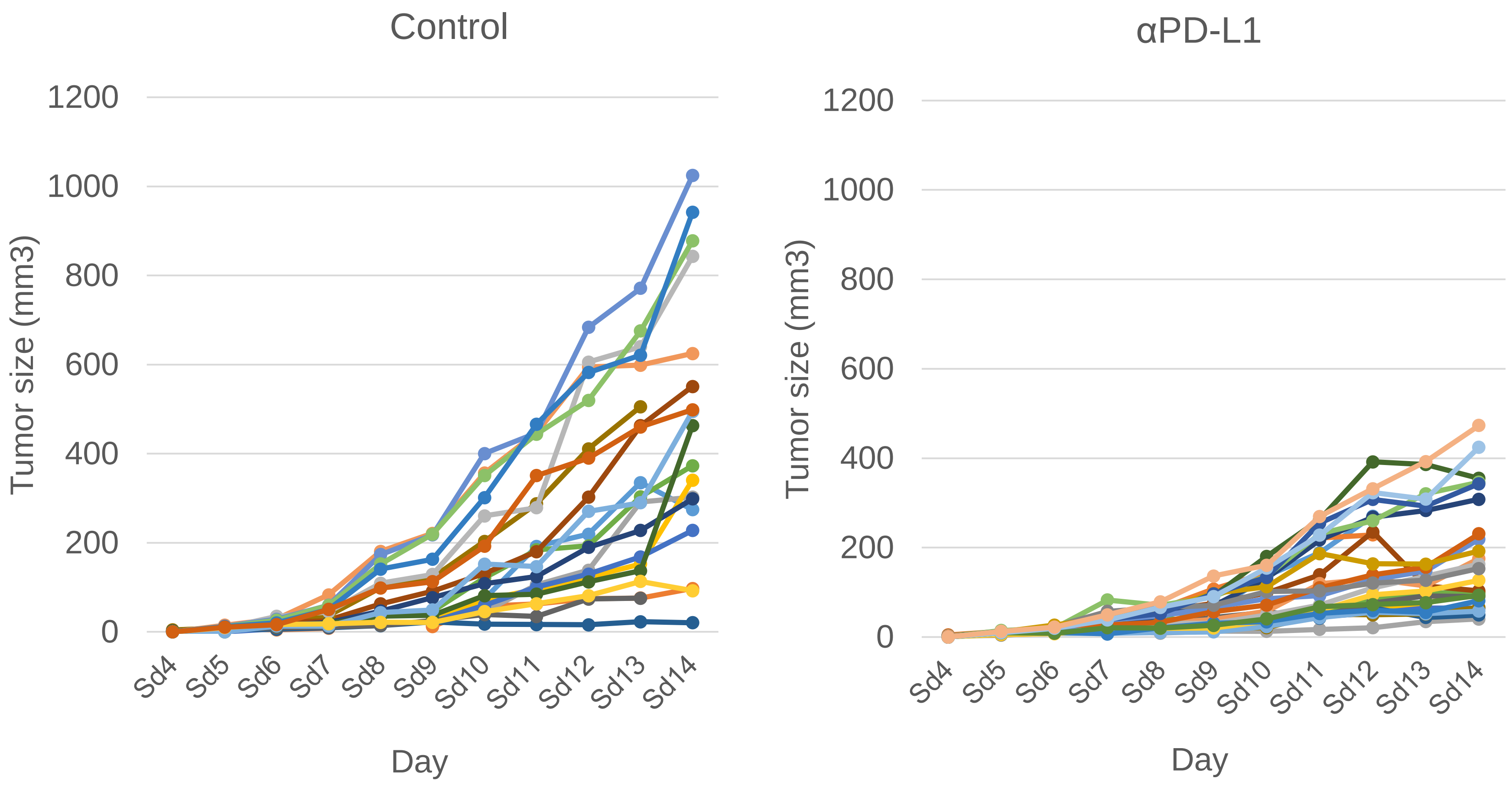

Figure S2. Individual tumor growth graph. It was confirmed for each individual that the tumor growth of ICI treatment group was inhibited compared to the control group.

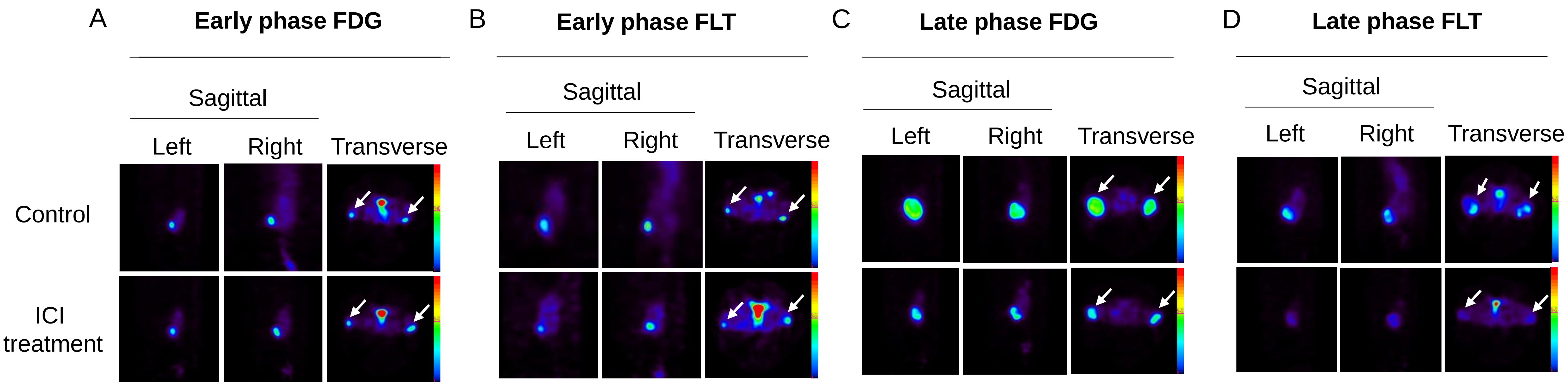

Figure S3. PET images of sagittal and transverse of each individual in the early and late phases of ICI treatment. (A) ^{18}F-FDG PET showed high TBR (Target to Background Ratio) on PET images (sagittal, transverse) in the ICI-treated group in the early phase , but ^{18}F-FLT PET showed no significant difference between the two groups (B). (C) ^{18}F-FDG PET in the late phase of ICI treatment showed with high TBR (sagittal, transverse) in both groups. (D) On the other hand, ^{18}F-FLT PET showed less tumor uptake in the ICI treatment group than in the control group. The white arrow indicates the tumor uptake lesion.
